# Emergency neurosurgery for traumatic brain injury by general surgeons at local hospitals in Sweden: a viable option when time is brain

**DOI:** 10.1186/s13049-024-01290-2

**Published:** 2024-11-15

**Authors:** Francisco Leal-Méndez, Lina Holmberg, Per Enblad, Anders Lewén, Fredrik Linder, Teodor Svedung Wettervik

**Affiliations:** 1 Department of Medical Sciences, Section of Neurosurgery, Uppsala University, Uppsala, SE-751 85 Sweden; 2 Department of Surgical Sciences, Section of Vascular Surgery, Uppsala University, Uppsala, SE-751 85 Sweden

**Keywords:** Craniotomy, Intracranial bleeding, Neurosurgery, Outcome, Traumatic brain injury

## Abstract

**Background:**

Timing of surgical evacuation of mass lesions in traumatic brain injury (TBI) is crucial. However, due to geographical variations, transportation time to the nearest neurosurgical department may be long. To save time, general surgeons at a local hospital may perform the operation, despite more limited experience in neurosurgical techniques. This study aimed to determine whether patient outcomes differed between those who had undergone emergency neurosurgery at local hospitals by general surgeons vs. at university hospitals by neurosurgeons.

**Methods:**

A nationwide observational study was performed using data from the Swedish Trauma Registry (SweTrau) between 2018 and 2022. A total of 565 TBI patients (local hospitals, *n* = 21; university hospitals, *n* = 544) who underwent intracranial hematoma evacuation within 8 h after arrival at the hospital were included. Data on demography, admission variables, traumatic injuries, and outcome (Glasgow Outcome Scale [GOS]) at discharge were evaluated. Favourable vs. unfavourable outcomes were defined as GOS scores of 4–5 vs. 1–3.

**Results:**

Compared with those treated at university hospitals, patients treated with intracranial hematoma evacuation at local hospitals had lower median Glasgow Coma Scale (GCS) scores (8 vs. 12, *p* < 0.001), higher rate of acute subdural hematomas (86% vs. 77%, *p* < 0.001), and lower rate of contusions (14% vs. 53%, *p* = 0.01). Being operated on at a local hospital was independently associated with higher mortality (*p* = 0.03) but with a similar rate of favourable outcome (*p* = 0.74) in multiple logistic regressions after adjustment for demographic and injury-related variables.

**Conclusions:**

Although a slightly greater proportion of patients who underwent emergency neurosurgery at local hospitals died, there was no difference in the rate of favourable outcome. Thus, in patients with impending brain herniation, when time is of the essence, evacuation of traumatic intracranial bleeding by general surgeons at local hospitals remains a highly viable option.

**Supplementary Information:**

The online version contains supplementary material available at 10.1186/s13049-024-01290-2.

## Introduction

Traumatic brain injury (TBI) is a common condition that contributes to significant mortality and morbidity worldwide [1–4]. In severe cases, patients may develop brain herniation syndromes due to expansive traumatic mass lesions [5–7]. Under these circumstances, access to urgent, life-saving surgery is of utmost importance to relieve the mass effect and restore cerebral perfusion [7–10]. Evacuation of intracranial bleeding is a frequent neurosurgical procedure, but the immediate availability to a neurosurgical unit for such care may be limited for several reasons. Some low-income countries do not have wide access to neurosurgical care [11]. Even in advanced healthcare systems, the geographical distance between a local hospital and a specialised university hospital may be large [12, 13], with transportation times of several hours, which may be too long to save the patient’s life. In this scenario, a dilemma arises: does the risk of transporting the patient to a neurosurgical facility, and therefore delaying surgical treatment, outweigh the risk of operating the patient immediately at the local hospital by general surgeons with less experience in neurosurgical technique? At present, only a handful of studies have investigated this issue [12–14], with mixed results. A smaller Norwegian study revealed a more dismal prognosis for those patients who underwent surgery by general surgeons, although this was to some extent confounded by TBI severity [13]. However, in other studies based on a region in central Sweden, our group reported that TBI patients who required emergency neurosurgery at local hospitals exhibited favourable outcome, comparable to those who were treated at the neurosurgical unit [12, 15]. The different results among the studies may be partly explained by variation in infrastructure, e.g., clinical decision-making regarding which patients should be operated on locally vs. immediately transported to a university hospital and how well prepared the general surgeons are for such an event (e.g., training programs in neurosurgical competence) [12–14].

There is currently a paucity of evidence to guide clinicians in this complex situation. Therefore, the aim of this nationwide, Swedish cohort study was to investigate variations in decision-making among the six regions within the country and whether there were differences in patient phenotypes and outcomes for those treated with emergency neurosurgery by general surgeons at local hospitals versus those treated by neurosurgeons at university hospitals. We hypothesised that emergency neurosurgery by general surgeons at local hospitals would be more common when the distance to a neurosurgical facility was far greater. We also hypothesised that those operated on at local hospitals would be in a worse neurological condition at arrival, necessitating such an exceptional event. However, after adjustment for this, the rate of functional outcome would be comparable to that of the patients treated by neurosurgeons.

## Materials and methods

### Patients and study design

**Fig. 1:**
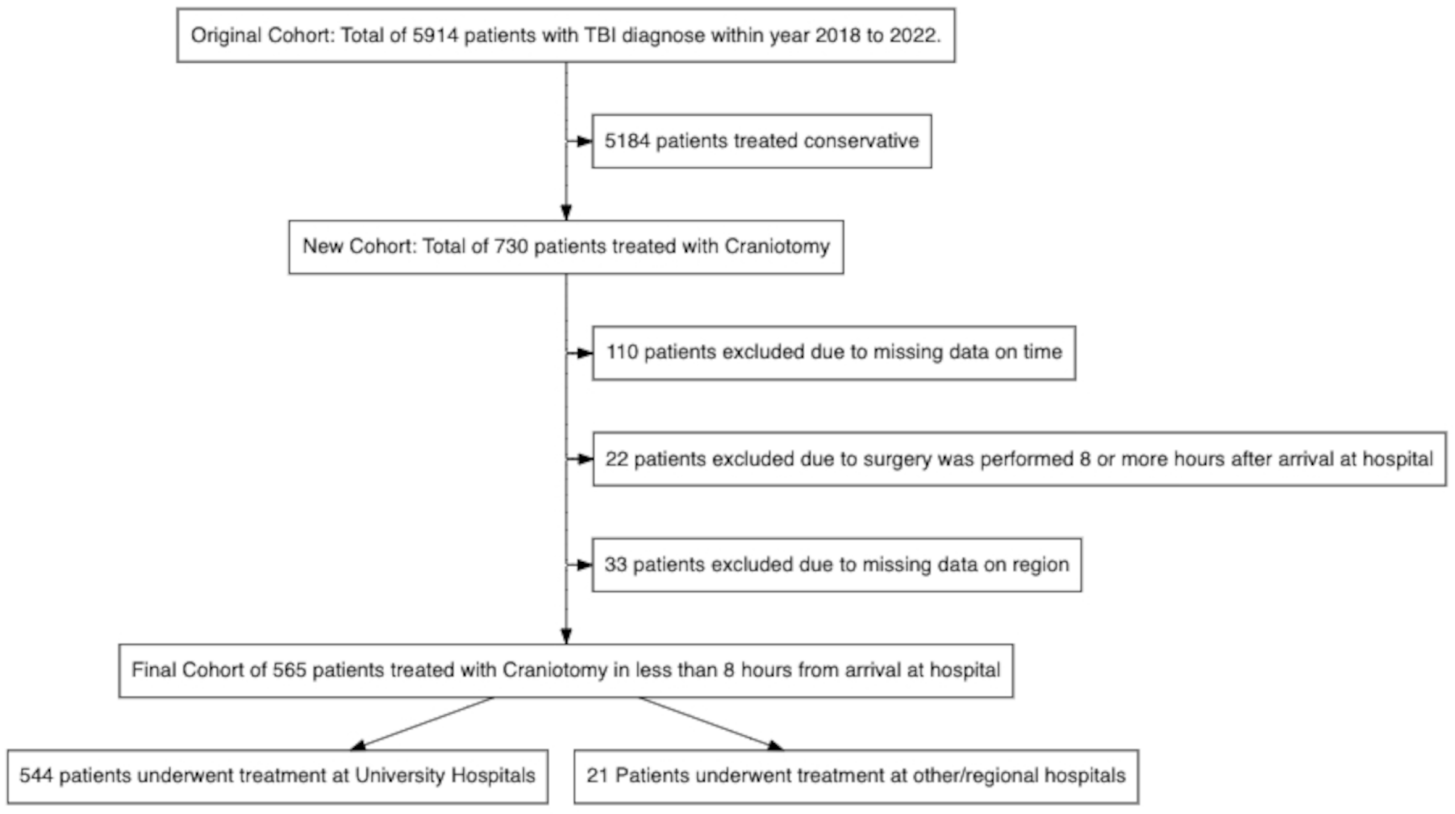
Flow chart of patient inclusion In this study, 5914 patients in the SweTrau register treated for TBI (ICD-10 codes S06.0 to S06.9) at any ICU in Sweden from the 1st of January 2018 to the 31st of December 2022 were eligible for inclusion, while those 5184 who were treated conservatively were excluded (Fig. 1). Furthermore, 165 patients were excluded: 110 with no data on time between arrival at hospital and surgery, 22 patients who underwent craniotomy more than 8 h post-arrival, and 33 without data on the treating hospital. Thus, the final study population included 565 TBI patients treated with craniotomy within 8 h post-arrival, 544 of them being operated on at a university hospital by neurosurgeons and 21 patients operated on at a local hospital by general surgeons without special neurosurgical training ICD = International Classification of Diseases. ICU = Intensive Care Unit. TBI = Traumatic Brain Injury

This was a multi-centre, retrospective study based on the SweTrau registry [16]. SweTrau includes all trauma patients with an activated trauma call or a New Injury Severity Score (NISS) > 15, which includes practically all trauma patients treated in intensive care units (ICUs) in Sweden. The registry has a completeness close to 89% and an accuracy of 86% [16]. A total of 5914 patients who had been treated due to TBI (ICD-10 codes S06.0 to S06.9) at any ICU in Sweden from the 1st of January 2018 to the 31st of December 2022 were eligible for inclusion, of whom 5184 conservatively treated patients (no craniotomy) were excluded (Fig. 1). Furthermore, 165 additional patients were excluded; 110 patients with no data on the time between arrival at the hospital and surgery, 22 patients who underwent craniotomy more than 8 h post-arrival, and 33 patients without data on the treating hospital. Thus, the final study population included 565 TBI patients treated with craniotomy within 8 h post-arrival, 544 of whom were operated on at a university hospital by neurosurgeons and 21 patients operated on at a local hospital by general surgeons.

### Swedish healthcare system and care trajectories in traumatic brain injury

**Fig. 2:**
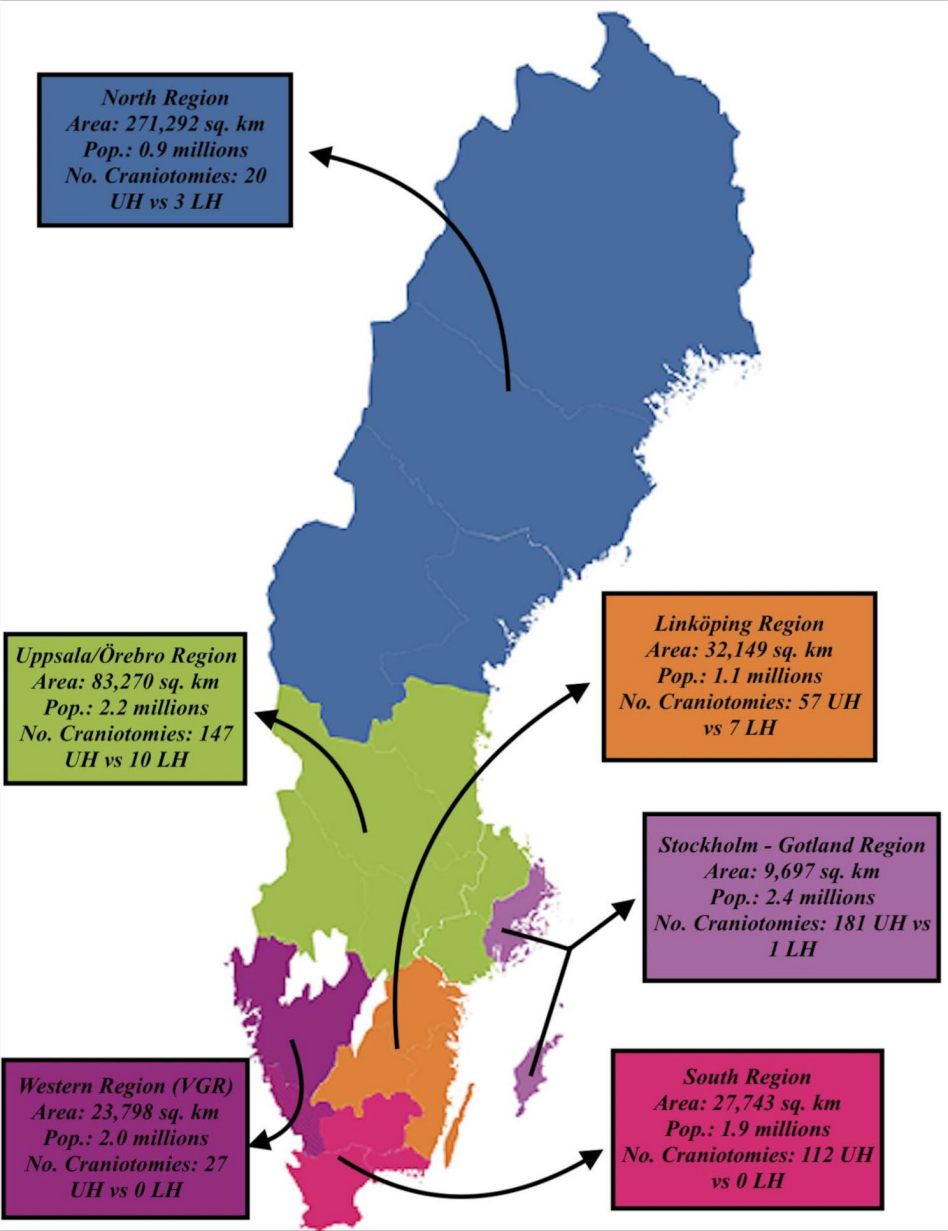
Map of Sweden divided into its six healthcare regions This map illustrates that surgical evacuation of traumatic intracranial bleedings was more often performed at local hospitals amongst regions with bigger geographical areas (e.g., Uppsala/Örebro, Linköping, and North Region) in comparison to more densely populated ones (Stockholm-Gotland, Western Region (VGR), and South Region) UH = University Hospital. LH = Local Hospital. TBI = Traumatic Brain Injury

The Swedish healthcare system is geographically and administratively divided into 6 major regions (Fig. 2). The catchment area and population size differ extensively among them. For example, the Stockholm-Gotland region includes the capital city of Sweden (Stockholm) and exhibit the largest population (approximately 2.5 million) but holds the smallest geographical area (9,697 km^2^), whereas the North Region serves the smallest patient population (approximately 0.9 million) but includes the largest geographical area (271,292 km^2^). Each region has several local hospitals and one university hospital that operates as tertiary centres for neurosurgical care. Only Region Uppsala/Örebro has *two* university hospitals (Uppsala and Örebro University hospitals) with neurosurgery (Uppsala and Örebro University hospital, respectively), where Uppsala receives trauma patients from the largest part of the catchment area. In small geographical regions such as Stockholm/Gotland, moderate to severe TBI patients are typically immediately transferred to university hospitals with specialised trauma and neurosurgical care. However, in regions with larger geographical areas, the vast majority of TBI patients are initially managed at local hospitals according to the ATLS concept [17] and are only transferred to university hospitals in severe cases that require neurosurgical procedures such as hematoma evacuation with craniotomy, invasive neuromonitoring, and neurointensive care. However, in some cases with traumatic intracranial bleeding, the patient may already exhibit clinical and radiological signs of impending brain herniation at the local hospital. In this scenario, the general surgeons at the local hospital discuss with the neurosurgical consultant at the university hospital whether surgery should be performed locally to save time or if the patient should be transferred to the neurosurgical facility. This decision could depend on the estimated transportation time between the local and university hospital as well as the experience of the general surgeon at the local hospital.

### Data acquisition

All data was retrieved from SweTrau [16], including demography (age, sex) and injury mechanisms (falls, road traffic accidents, etc.), as well as descriptors of the clinical condition (Glasgow Coma Scale [GCS], abbreviated injury score [AIS] head [18], injury severity score [ISS] [19]), intracranial injuries (epidural hematoma [EDH], acute subdural hematoma [ASDH], traumatic subarachnoid hemorrhage [tSAH], and contusions) and treatment (craniotomy). The outcome was evaluated via the Glasgow Outcome Scale (GOS) at discharge from the hospital [20], ranging from 1 (death) to 5 (good recovery), and then divided into mortality vs. survival (GOS = 1 vs. GOS 2 to 5) and favourable vs. unfavourable (GOS 4 to 5 vs. 1 to 3).

### Statistical analysis

Demography, admission status, injury mechanism, time to surgery, diagnosis, and clinical outcome were described as median (interquartile range) or number (proportion). The Mann-Whitney U-test and Pearson’s Chi-square analyses were used for statistical comparisons of these variables between patients who underwent craniotomy in less than 8 h from arrival at a university hospital by a neurosurgeon vs. a local hospital by a general surgeon. Missing values were excluded from the analyses. Multiple logistic regression analyses were performed to determine if craniotomy at a university or local hospital had an independent association with mortality and favourable outcome, respectively, after adjustment for age, GCS on arrival, ISS, ASDH (yes/no), and contusions (yes/no). The baseline variables were chosen to take into account demography (age), injury severity (GCS and ISS), and differences in traumatic injury patterns between the patients operated on at a local vs. a university hospital (ASDH and contusions – as seen in the univariate analysis). A *p*-value < 0.05 was considered statistically significant. The statistical analyses were conducted in RStudio software (version 2024.04.1).

## Results

### Catchment areas and population in relation to craniotomy surgery

A total of 565 patients were operated on in less than 8 h from their arrival at the hospital (Table 1). These patients were further divided into two groups, the first with 544 (96%) patients that underwent craniotomy at a university hospital and a second group with 21 (4%) patients that were operated with craniotomy at a local hospital. A total of 338 patients of the first group were initially admitted to another hospital before being transferred for definitive treatment with craniotomy at a university hospital (62%). Thus, the remainder of the group (206 patients, 38%) arrived directly to the emergency department of the university hospital. There was some regional variation in the distribution of craniotomy surgery at local hospitals. As demonstrated in Fig. 2, fewer craniotomies were performed at local hospitals in regions with smaller geographical area regardless of the craniotomy rate per hundred thousand inhabitants/year. The Stockholm – Gotland region, which has the smallest area and the highest rate of craniotomies per hundred thousand inhabitants/year (1.5), had only one patient operated on at a local hospital in Gotland, with limited possibilities of patient transport without a helicopter. The more densely populated regions, Western Region (VGR) and South Region, with craniotomy rates of 0.3 and 1.2 respectively, had no cases operated on at local hospitals. In contrast, 95% of the patients operated on at local hospitals lived in one of the three geographically largest regions, including Uppsala/Örebro, Linköping, and North Region.

**Table 1:** Catchment areas, populations, patients, and treatment pathways for each neurosurgical region

Region	Catchment area (millions)*	Surface Area (km^2^)	Population density (inhabitants per km^2^)*	TBI patients in total, *n* (%)	Patients treated with craniotomy (regardless of time), *n* (%)	Patients treated with craniotomy < 8 h at a University hospital, *n* (%)	Patients treated with craniotomy < 8 h at local hospitals, *n* (%)
South Region	1.9 (18%)	27,743	68	872 (15%)	127 (18%)	112 (21%)	0 (0%)
Western Region	2.0 (19%)	23,798	70	700 (12%)	62 (9%)	27 (5%)	0 (0%)
Linköping Region	1.1 (10%)	32,149	34	630 (11%)	83 (12%)	57 (10%)	7 (33%)
Stockholm – Gotland Region	2.4 (24%)	9,697	377	1801 (32%)	191 (27%)	181 (33%)	1 (5%)
Uppsala/Örebro Region	2.2 (20%)	83,270	33	1349 (24%)	203 (29%)	147 (27%)	10 (48%)
North Region	0.9 (9%)	271,292	4.5	296 (5%)	31 (4%)	20 (4%)	3 (14%)
Total	10.5 (100%)	447,949	26***	5648 (100%)	697 (100%)**	544 (100%)	21 (100%)

*Data from the Swedish Central Bureau of Statistics (SCB). Data by december 31st 2022

** Missing data: 33 patients with no data on region

*** Population density for Sweden taken directly from the Swedish central bureau of statistics (SCB), it is not a mean of the region’s population density

### Demography, injuries, and treatments – local vs. university hospitals

As demonstrated in Table 2, the patients who underwent craniotomy within 8 h of arrival were aged around 50 years old and the majority (around 70%) were males in both the cohort operated on at the university and the local hospitals. The latter group exhibited a worse neurological status in the emergency department, with a lower median GCS at 8 (IQR 4–12) compared to 12 (IQR 8–14) at the university hospitals (*p* < 0.001). Otherwise, both groups had a similar AIS head and ISS score. Furthermore, the patients operated on at a local hospital more often had an ASDH (86% vs. 77%, *p* < 0.001) and less often contusions (14% vs. 53%, *p* = 0.01) than those treated at a university hospital. The median time from arrival to surgery were slightly below 2 h in both groups. Descriptive data for each centre are demonstrated for those operated on at a university hospital (Supplementary Table 1) and at a local hospital (Supplementary Table 2). Moreover, in a sub-analysis of (i) those admitted and operated on at the local hospital, (ii) those admitted at the local hospital, immediately transferred to a university hospital for craniotomy, and (iii) those admitted and operated on at a university hospital (Supplementary Table 3), the former group exhibited the worst neurological injury (lowest GCS), while those transferred exhibited a better neurological status (at least initially), and those admitted directly to a university hospital were in between those groups.

**Table 2:** Demography, injury status, treatments, and outcome – comparison between patients operated on at a University vs. Local Hospital

Variables	Patients treated with craniotomy < 8 h at a university hospital	Patients treated with craniotomy < 8 h at a local hospital	*p*-value
Patients, *n* (%)	544 (96%)	21 (4%)	Not applicable
Age (years), median (IQR)	51 (29–67)	55 (33–73)	0.50
Sex (male/female), *n* (%)	388/156 (71%/29%)	15/6 (71%/29%)	1.00
Injury mechanism, *n* (%)	Road = 135 (25%) Fall = 298 (55%) Blunt = 71 (13%) Penetrating = 10 (2%) Explosion = 1 (0%) Unknown = 29 (5%)	Road = 4 (19%) Fall = 14 (67%) Blunt = 2(10%) Penetrating = 0 (0%) Explosion = 0 (0%) Unknown = 1 (4%)	0.92
GCS at admission, median (IQR)	12 (8–14)	8 (4–12)	***< 0.001***
AIS head, median (IQR)	3 (3–4)	3 (2–5)	0.41
ISS, median (IQR)	26 (25–29)	26 (25–29)	0.34
Epidural hematoma, *n* (%)	178 (33%)	7 (33%)	1.00
Acute subdural hematoma, *n* (%)	420 (77%)	18 (86%)	***< 0.001***
Traumatic subarachnoid hemorrhage, *n* (%)	241 (44%)	10 (48%)	0.85
Contusion, *n* (%)	288 (53%)	3 (14%)	***0.01***
Time to surgery post-arrival (hours), median (IQR)	1.7 (0.9–2.8)	1.6 (1.0-2.4)	0.79
GOS, median (IQR)	3 (3–3)	3 (1–3)	0.14
Favourable/unfavourable**, *n* (%)	72/472 (13%/87%)	5/16 (24%/76%)	0.29
Mortality, *n* (%)	71 (13%)	9 (43%)	***<0.001***

*Missing data (both cohorts combined): Injury Mechanism (n) = 29; GCS (n) = 316; GOS (n) = 0; Mortality (n) = 3

** Favourable/unfavourable GOS = 4–5/1–3

Bold and italics indicate statistical significance

AIS = Abbreviated injury scale. GCS = Glasgow Coma Scale. GOS = Glasgow Outcome Scale. IQR = Interquartile range. ISS = Injury severity score

### Mortality and favourable outcome – local versus university hospitals

**Fig. 3:**
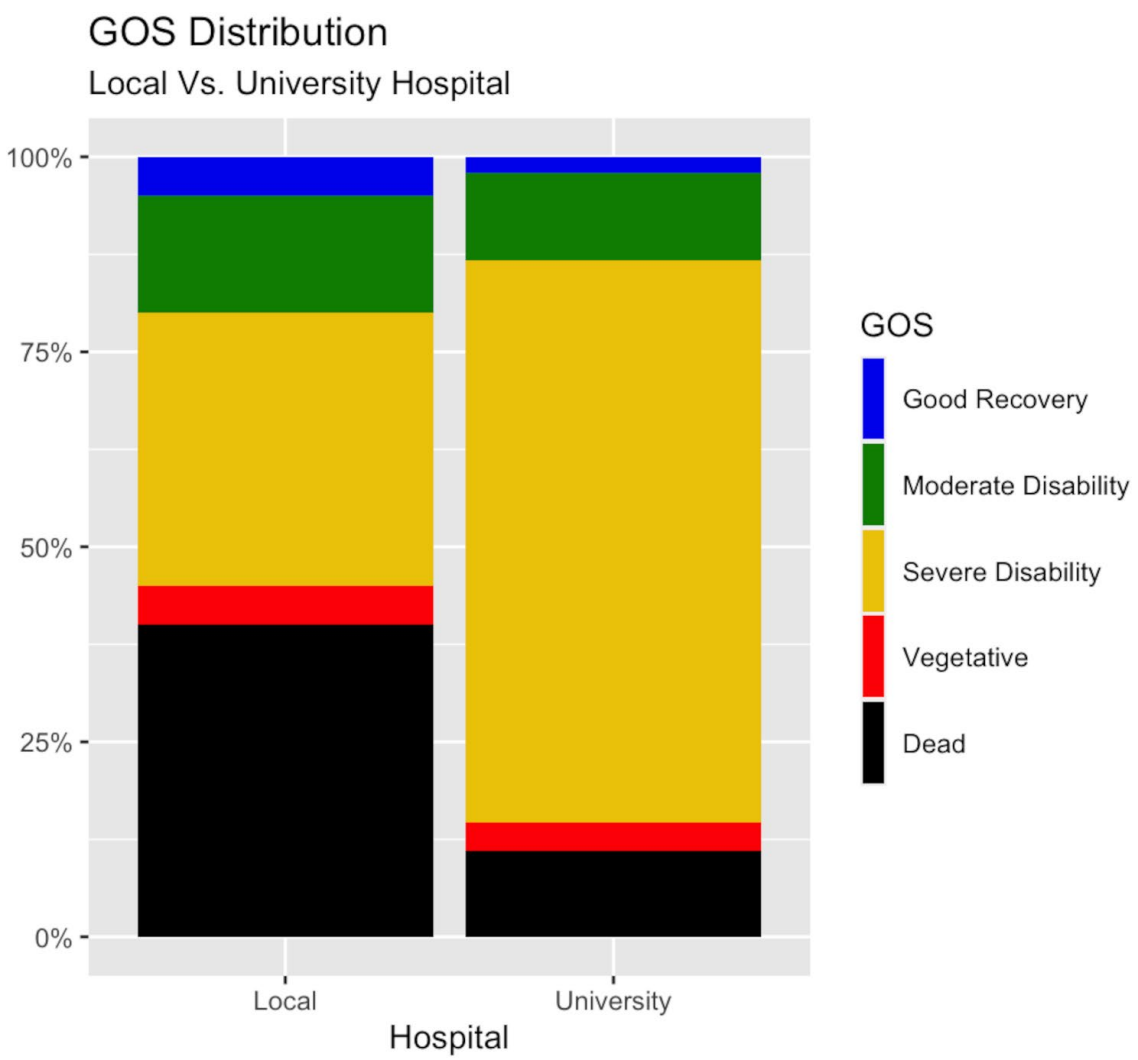
GOS distribution following TBI with craniotomy within 8 h – local vs. university hospital Distribution according to GOS at discharge for the population of patients operated at a local hospital by general surgeons in less than 8 h post-arrival (left) and the population that underwent craniotomy in less than 8 h post-arrival at a university hospital by neurosurgeons (right) for TBI. The patients treated at local hospitals exhibited a higher mortality, but there was no difference in the rate of favourable. outcome or median GOS at discharge as compared with those treated at a university hospital GOS = Glasgow Outcome Scale. TBI = Traumatic Brain Injury

According to the univariate analysis, the median GOS at discharge was 3 and did not significantly differ between those treated with craniotomy at a local hospital and those treated with craniotomy at a university hospital (Table 2; Fig. 3). Furthermore, the rate of favourable outcome was slightly, but not significantly higher (24% vs. 13%, *p* = 0.29), whereas the mortality rate was significantly higher (43% vs. 13%, *p* < 0.001) in the patient group operated on at a local rather than a university hospital. After adjustment for baseline variables (age, GCS, ISS, ASDH, and contusions), surgery at a university hospital remained significantly associated with lower mortality (OR (95% CI) = 0.16 (0.03–0.86), *p* = 0.03), while there was no independent association with favourable outcome (Table 3).

**Table 3:** Craniotomy at local vs. university hospital related to mortality and favourable outcome – multivariable logistic regression analysis

Variable	Mortality		Favourable outcome	
	OR (CI 95%)	*P* value	OR (CI 95%)	*P* value
Age (years)	1.05 (1.02–1.08)	***< 0.001***	0.96 (0.95–0.98)	***< 0.001***
GCS	0.85 (0.77–0.95)	***0.002***	1.11 (1.02–1.23)	***0.03***
ISS	1.04 (0.97–1.11)	0.24	0.95 (0.90–1.00)	0.08
Acute Subdural Hematoma	1.53 (0.41–7.10)	0.55	0.73 (0.34–1.60)	0.58
‍Contusion	1.73 (0.71–4.31)	0.23	0.38 (0.18–0.77)	***0.009***
Hospital (University)	0.16 (0.03–0.86)	***0.03***	1.33 (0.29–9.70)	0.74

Mortality: AIC = 163.9; *r*^2^ = 0.21; AUROC (95% CI) = 0.84 (0.74–0.94)

Favourable outcome: AIC = 229.3; *r*^2^ = 0.20; AUROC (95% CI) = 0.76 (0.62–0.90)

Bold and italics indicate statistical significance

AIC = Akaike Information Criteria. AUROC = Area Receiver Operating Characteristics Curve. CI = Confidence Interval. GCS = Glasgow Coma Scale. ISS = Injury severity score. OR = Odds Ratio

## Discussion

In this nationwide cohort study of 565 Swedish TBI patients who required emergency neurosurgery over a 5-year period, a small portion (only 4%) were treated by general surgeons at local hospitals rather than neurosurgeons at university hospitals. Most of the former cases were operated on in the Swedish regions with the largest geographical areas and the longest distances to a neurosurgical facility. The patients treated at local hospitals were in a worse neurological condition at arrival. These patients exhibited increased mortality, even after adjustment for injury severity and TBI subtype, but there was no difference in the rate of favourable outcome compared to those treated by neurosurgeons. Thus, while the surgical result may be slightly worse in less experienced hands, we argue that the outcome was overall favourable, given the alternative to wait up to several hours due to transportation to a neurosurgical facility. Altogether, we think that emergency neurosurgery performed by general surgeons at local hospitals is a viable option, particularly in cases with impending brain herniation and a long distance to the nearest neurosurgical facility.

First, there was a clear difference in the rate of emergency neurosurgery at local hospitals among the six regions over the study period. The university hospitals in Stockholm/Gotland, Western Region and South Region take care of the three most populated cities in Sweden and encompass relatively smaller geographical areas. On the contrary, the university hospitals in Uppsala/Örebro, Linköping, and North Region take care of many smaller cities and encompass relatively large geographical areas. In the group treated with emergency neurosurgery by general surgeons, only one (5%) of these cases was done in the former regions with smaller geographical areas and the remaining 20 (95%) in the latter regions. Thus, as expected, emergency neurosurgery at local hospitals was mostly considered in regions with a long distance/transportation time between the local hospital and a neurosurgical facility. Furthermore, the decision to proceed with emergency neurosurgery was likely also related to tradition and infrastructure. As a consequence of the relatively large geographical area, the Uppsala/Örebro region has a long experience with training general surgeons at local hospitals in neurosurgical technique to achieve a sufficient level of preparedness for these events [12]. This explains why Uppsala/Örebro had the largest proportion of emergency neurosurgeries performed at local hospitals.

Second, the demographic and injury variables clearly showed that those operated on at local hospitals were in a neurologically worse condition, with lower GCS at arrival. This group also had a higher rate of ASDH and lower rate of contusions than those operated on by neurosurgeons. These findings suggest that there had to be a more urgent indication (worse neurological status) that tilted the scale towards proceeding with emergency neurosurgery locally, prioritising a shorter time to resolution as more beneficial to the patient at the expense of it being performed by less experienced hands. Emergency neurosurgery by general surgeons was also focused on evacuation of extracerebral lesions, i.e., not contusions, as the latter lesion type typically requires more technical expertise and is performed only by neurosurgeons. Unfortunately, SweTrau does not provide more detailed data regarding symptoms and imaging variables indicative of brain herniation (pupillary reactivity and extent of mass effect) nor regarding the specific type of lesion that was evacuated. This constitutes a source of bias which the authors believe should be acknowledged.

Third, the patients who had been operated on at local hospitals had a similar median GOS and rate of favourable outcome at discharge as those treated by neurosurgeons. However, mortality was higher in the former group, even after adjustment for demography and injury severity. Although we proceeded with multiple logistic regressions to adjust for confounders, important prognostic factors such as pupillary reactivity [21] and more detailed descriptors of the mass lesions [22] were not available and could not be taken into account in these analyses. We expect that the group that underwent surgery at local hospitals more often exhibited unreactive pupils and worse mass lesions due to selection bias, i.e., if they exhibited signs of impending brain herniation, it was more likely that the responsible physician would consider that the patient would not tolerate a treatment delay. However, the results probably also reflect that surgical outcome is not as favourable in less experienced hands. Nevertheless, the question is not whether patients should be operated on by general surgeons or neurosurgeons in general but rather if emergency neurosurgery at local hospitals by general surgeons is a better alternative than delaying definitive therapy by several hours due to the need of transporting patients with space occupying lesions to a neurosurgical facility. Taking into account the rapid course of brain herniation, the detrimental effect of a longer time to treatment in this scenario [7–10, 23, 24], and the comparable rates of favourable outcome in the cohorts operated on at local vs. university hospitals, we believe that emergency neurosurgery by general surgeons is a viable option in severe TBI cases, after careful decision-making based on the urgency of surgery, geographical/logistic conditions, and experience at the local hospital. We may add, to reinforce this notion, that the worst ratio of favourable to unfavourable outcome in this study was observed in the group of patients that were first admitted to a local hospital and then transported to a university hospital for surgical resolution (Supplementary Table 3). It is also important to factor in, although not being the primary focus of this study, the impact that transporting these patients may have. We noticed that 41 patients, or close to 12% of those in the group that was directly taken to a university hospital for surgical treatment (Supplementary Table 3), did so via a helicopter ambulance. Even though the helicopter provides a faster transportation, it may not always be available due to logistic (e.g., weather conditions) and economic factors.

### Methodological considerations

The main strength of this study is the nationwide design using the SweTrau registry, which has a near complete coverage of trauma patients treated in intensive care units in Sweden [16]. There are also some limitations. The total number of patients treated with emergency neurosurgery at local hospitals was relatively small, which reduces the reliability of our results to some extent. Inclusion of all transferred patients operated within 8 h after arrival to the university hospital in the control group is associated with some methodological issues. Some of those patients, in regions practicing life-saving evacuations in local hospitals, may have been judged not to have a life-threatening condition requiring immediate surgery, while such patients may represent a mix of life-threatening conditions and less acute injuries in regions where no neurosurgical operations are performed in local hospitals. However, the results were virtually the same when the comparison was made with only patients arriving directly to the university hospitals (Supplementary Table 3).

Furthermore, although the registry contains many important variables related to trauma severity, we were unable to investigate details about preoperative pupillary reactivity, the extent of mass effect, and which specific intracranial lesion was evacuated. This fact impeded us from performing more in-depth investigations into the presence of brain herniation preoperatively, the urgency to proceed with emergent treatment, and what these surgeries entailed. This limits the impact of these results. Additionally, GOS was evaluated at discharge, i.e., a relatively short time after the trauma as compared to 6 to 12 months post-injury when the patients would have had more time to recover. This contributed to a high proportion of patients classified with unfavourable outcome. Still, an early outcome estimation may be useful to better evaluate the effects of early interventions, such as emergency neurosurgery, while a later measurement would be expected to also reflect the extent of neurorehabilitation and re-integration into society. In addition, the external validity of our results may be limited, as it depends on a specific geography, the organisation of healthcare, and the tradition of neurosurgical training of general surgeons at local hospitals.

## Conclusions

In this nationwide cohort study on TBI patients who required emergency neurosurgery, only a small proportion were treated by general surgeons at local hospitals rather than neurosurgeons at university hospitals. The majority of surgeries at local hospitals occurred in those Swedish regions with the longest distance to the neurosurgical department at a university hospital. These patients were in a worse neurological condition at arrival. They also exhibited a higher mortality, even after adjustment for injury severity and TBI subtype, but there was no difference in the rate of favourable outcome. Thus, although the general surgeons at local hospitals had less experience in neurosurgical technique, we still consider their results as favourable, especially in light of the alternative, i.e., delaying treatment by several hours due to transportation to a neurosurgical facility. Thus, while every individual case requires careful decision-making, we think that emergency neurosurgery performed by general surgeons at local hospitals is a viable option in severe cases when time is brain.

## Electronic supplementary material

Below is the link to the electronic supplementary material.

**Supplementary Table 1**: Descriptive data about patients that underwent surgery within 8 hours at University Hospital by region. **Supplementary Table 2**: Descriptive data about patients who underwent surgery within 8 hours at Local Hospital by region. **Supplementary Table 3**: Data about patients operated within 8 hours - Local vs. University hospitals (transferred vs. directly admitted)

## Data Availability

The data are available upon reasonable request.

## Abbreviations

TBI: Traumatic Brain Injury
SweTrau: Swedish Trauma Registry
GOS: Glasgow Outcome Scale
GCS: Glasgow Coma Scale
NISS: New Injury Severity Score
ICUs: Intensive Care Units
AIS: Abbreviated Injury Score
ISS: Injury Severity Score
EDH: Epidural Hematoma
ASDH: Acute Subdural Hematoma
OR: Odds Ratio
CI: Confidence Interval
VGR: Western Region
IQR: Interquartile Range
ICD: International Classification of Diseases
UH: University Hospital
LH: Local Hospitals
SCB: Swedish Central Bureau of Statistics
AIC: Akaike Information Criteria
AUROC: Area Under the Receiver Operating Characteristics Curve
