# Supplementary material for: Emergency neurosurgery for traumatic brain injury by general surgeons at local hospitals in Sweden: a viable option when time is brain
Source: Scand J Trauma Resusc Emerg Med. 2024 Nov 15;32:115. doi: 10.1186/s13049-024-01290-2 (PMC11568597; doi:10.1186/s13049-024-01290-2)
Supplement: Supplementary file 1 — Supplementary Table 1: Descriptive data about patients that underwent surgery within 8 hours at University Hospital by region. Supplementary Table 2: Descriptive data about patients who underwent surgery within 8 hours at Local Hospital by region. Supplementary Table 3: Data about patients operated within 8 hours - Local vs. University hospitals (transferred vs. directly admitted) [file 13049_2024_1290_MOESM1_ESM.docx]

**Supplementary Table 1. Descriptive data about patients that underwent surgery within 8 hours at University Hospital by region.**

| Variables | Patients treated with craniotomy < 8 h at the Neurosurgery department | Stockholm – Gotland Region | Uppsala/Örebro Region | Linköping Region | South Region | Western Region | North Region |
| --- | --- | --- | --- | --- | --- | --- | --- |
| Patients, n (%) | 544 (100%) | 181 (33%) | 147 (27%) | 57 (10%) | 112 (21%) | 27 (5%) | 20 (4%) |
| Age (years), median (IQR) | 51 (29-67) | 48 (29 -63) | 51 (29-68) | 49 (27-65) | 59 (39-72) | 29 (19-52) | 53 (33-70) |
| Sex (male/female), n (%) | 388/156 (71%/29%) | 137/44 (76%/24%) | 107/40 (73%/27%) | 38/19 (67%/33%) | 72/40 (64%/36%) | 21/6 (78%/22%) | 13/7 (65%/35%) |
| GCS at admission, median (IQR) | 12 (8-14) | 12 (8-14) | 10 (7-15) | 14 (10-15) | 13 (10-14) | 13 (3-14) | 10 (6-14) |
| ISS, median (IQR) | 26 (25-29) | 26 (25-30) | 25 (25-29) | 25 (21-26) | 26 (25-27) | 26 (25-33) | 26 (22-29) |
| Epidural hematoma, n (%)* | 178 (33%) | 65 (37%) | 43 (24%) | 16 (9%) | 33 (19%) | 13 (7%) | 8 (4%) |
| Acute subdural hematoma, n (%) | 420 (77%) | 144 (34%) | 112 (27%) | 43 (10%) | 89 (22%) | 18 (4%) | 14 (3%) |
| Traumatic subarachnoid hemorrhage, n (%) | 241 (44%) | 88 (37%) | 56 (23%) | 18 (7%) | 57 (24%) | 9 (4%) | 13 (5%) |
| Contusion, n (%) | 288 (53%) | 106 (36%) | 68 (24%) | 24 (8%) | 64 (22%) | 13 (5%) | 13 (5%) |
| Time to surgery post-arrival (hours), median (IQR) | 1.67 (0.93-2.83) | 1.67 (1.67-2.55) | 1.92 (1.03-3.28) | 1.58 (0.82-3.32) | 1.20 (0.63-2.56) | 1.50 (1.13-2.27) | 2.13 (1.66-2.90) |
| GOS, median (IQR) | 3 (3-3) | 3 (3-3) | 3 (3-3) | 3 (3-3) | 3 (3-3) | 3 (3-4) | 3 (3-3) |
| Favourable/Unfavourable**, n (%) | 72/472 (13%/87%) | 27/154 (15%/85%) | 20/127 (14%/86%) | 5/52 (9%/91%) | 9/103 (8%/92%) | 9/18  (33%/67%) | 2/18 (10%/90%) |
| Mortality, n (%) | 71 (13%) | 25 (35%) | 10 (14%) | 7 (10%) | 22 (31%) | 7 (10%) | 0 (0%) |

*Percentage of patients that had “Epidural Hematoma” as diagnosis in the University hospital Cohort as a whole. Applies even for “Acute Subdural Hematoma”, “Traumatic Subarachnoid Hemorrage” and “Contusion”.

**Favourable/unfavourable GOS = 4-5/1-3. Isolated percentage values per region.

**Supplementary Table 2. Descriptive data about patients who underwent surgery within 8 hours at Local Hospital by region.**

| Variables | Patients treated with craniotomy < 8 h by general surgeons at local hospitals | Stockholm – Gotland Region | Uppsala/Örebro Region | Linköping Region | South Region | Western Region | North Region |
| --- | --- | --- | --- | --- | --- | --- | --- |
| Patients, n (%) | 21 (4%) | 1 (5%) | 10 (48%) | 7 (33%) | 0 (0%) | 0 (0%)0 0 | 3 (14%) |
| Age (years), median (IQR) | 55 (33-73) | 59 (N/A) | 60 (51-75) | 33 (19-46) | N/A | N/AN/A | 76 (66-78) |
| Sex (male/female), n (%) | 15/6 (71%/29%) | 1/0 (100%/0%) | 7/3 (70%/30%) | 5/2 (71%/29%) | N/A | N/A | 2/1 (67%/33%) |
| GCS at admission, median (IQR) | 8 (4-12) | 3 (N/A) | 8 (5-13) | 9 (7-11) | N/A | N/A | 3 (3-3) |
| ISS, median (IQR) | 26 (25-29) | 25 (N/A) | 26 (25-27) | 29 (26-29) | N/A | N/A | 27 (17-29) |
| Epidural hematoma, n (%)* | 7 (33%) | 0 (0%) | 1 (14%) | 5 (72%) | N/A | N/A | 1 (14%) |
| Acute subdural hematoma, n (%) | 18 (86%) | 1 (6%) | 10 (56%) | 4 (22%) | N/A | N/A | 3 (16%) |
| Traumatic subarachnoid hemorrhage, n (%) | 10 (48%) | 0 (0%) | 6 (60%) | 3 (30%) | N/A | N/A | 1 (10%) |
| Contusion, n (%) | 3 (14%) | 0 (0%) | 1 (33%) | 2 (67%) | N/A | N/A | 0 (0%) |
| Time to surgery post-arrival (hours), median (IQR) | 3.16 (N/A) | 1.79 (0.88-2.34) | 1.61 (0.99-2.54) | 1.60 (1.35-1.65) | N/A | N/A | 1.23 (0.68-3.33) |
| GOS, median (IQR) | 3 (1-3) | 3 (3-3) | 1 (1-3) | 3 (2-4) | N/A | N/A | 3 (1-4) |
| Favourable/Unfavourable**, n (%) | 5/16 (24%/76%) | 0/1 (0%/100%) | 1/9 (10%/90%) | 3/4 (43%/57%) | N/A | N/A | 1/2 (33%/67%) |
| Mortality, n (%) | 9 (43%) | 0 (0%) | 6 (67%) | 2 (22%) | N/A | N/A | 1 (11%) |

*Percentage of patients that had “epidural hematoma” as diagnosis in the Local Hospital Cohort as a whole. Applies even for “Acute Subdural Hematoma”, “Traumatic Subarachnoid Hemorrage” and “Contusion”.

**Favourable/unfavourable GOS = 4-5/1-3. Isolated percentage values per region.

**Supplementary Table 3.** **Data about patients operated within 8 hours - Local vs. University hospitals (transferred vs. directly admitted)**

| **Variables** | **Patients treated with craniotomy < 8 h by general surgeons at local hospitals.** | **Patients Treated with craniotomy < 8 h by Neurosurgeons directly at University Hospital** | **Patients treated with craniotomy in < 8 h by Neurosurgeons at University Hospital after being transferred from Local Hospital** |
| --- | --- | --- | --- |
| Patients, n (%) | 21 (4%) | 206 (38%) | 338 (62%) |
| Age (years), median (IQR) | 55 (33-73) | 42 (24-64) | 55 (35-68) |
| Sex (male/female), n (%) | 15/6 (71%/29%) | 153/53 (74%/26%) | 235/103 (70%/30%) |
| Injury mechanism, n (%) | Road = 4 (19%);  Fall = 14 (67%);  Blunt = 2 (10%);  Penetrating = 0 (0%)  Explosion = 0 (0%)  Unknown = 1 (4%) | Road = 68 (33%);  Fall = 97 (47%);  Blunt = 27 (13%);  Penetrating = 8 (4%)  Explosion = 1 (0%)  Unknown = 5 (3%) | Road = 67 (20%);  Fall = 201 (59%);  Blunt = 44 (13%);  Penetrating = 2 (1%)  Explosion = 0 (0%)  Unknown = 24 (7%) |
| GCS at admission, median (IQR) | 8 (4-12) | 10 (6-13) | 14 (13-15) |
| AIS head, median (IQR) | 3 (2-5) | 3 (3-4) | 3 (3-4) |
| ISS, median (IQR) | 26 (25-29) | 26 (25-30) | 25 (25-27) |
| Epidural hematoma, n (%) | 7 (33%) | 72 (35%) | 106 (31%) |
| Acute subdural hematoma, n (%) | 18 (86%) | 155 (75%) | 265 (78%) |
| Traumatic subarachnoid hemorrhage, n (%) | 10 (48%) | 115 (56%) | 126 (37%) |
| Contusion, n (%) | 3 (14%) | 122 (59%) | 166 (49%) |
| Time to surgery post-injury (hours), median (IQR) | 1.6 (1.0-2.4) | 1.75 (1.25-2.70) | 1.6 (0.75 – 2.85) |
| GOS, median (IQR) | 3 (1-3) | 3 (3-3) | 3 (3-3) |
| Favourable/unfavourable**, n (%) | 5/16 (24%/76%) | 40/166 (19%/81%) | 32/306 (9%/91%) |
| Mortality, n (%) | 9 (43%) | 39 (19%) | 32 (9%) |

*Missing data (both cohorts combined): Injury Mechanism (n) = 29; GCS (n) = 316; GOS (n) = 0; Mortality (n) = 3.

** Favourable/unfavourable GOS = 4-5/1-3.

AIS = Abbreviated injury scale. GCS = Glasgow Coma Scale. GOS = Glasgow Outcome Scale. IQR = Interquartile range. ISS = Injury severity score.
